# VALIDATION OF COGNITIVE AND PSYCHOSOCIAL INSTRUMENTS FOR THE KENYAN CONTEXT: RESULTS FROM LOSHAK

**DOI:** 10.1093/geroni/igae098.2079

**Published:** 2024-12-31

**Authors:** Sneha Mani, Joshua Ehrlich, Anthony Ngugi, Niranjani Nagarajan, Roselyter Riang’a, Eunice Mwangi, Alden Gross

**Affiliations:** Johns Hopkins Bloomberg School of Public Health, Baltimore, Maryland, United States; University of Michigan, Ann Arbor, Michigan, United States; Aga Khan University, Nairobi, Nairobi Area, Kenya; Michigan Medicine, Ann Arbor, Michigan, United States; Aga Khan University, Nairobi, Nairobi Area, Kenya; Aga Khan University, Nairobi, Nairobi Area, Kenya; Johns Hopkins Bloomberg School of Public Health, Baltimore, Maryland, United States

## Abstract

Adaptation of cognitive tests to culturally diverse and low-resource settings is essential for expanding knowledge of cognitive health in older adults beyond high-income countries. However, contextual differences in novel settings can affect reliability and validity. A significant knowledge gap remains in Sub-Saharan Africa, a region projected to be home to over 158 million older adults by 2050. The Longitudinal Study of Health and Ageing in Kenya (LOSHAK) aims to address this measurement gap. The LOSHAK pilot collected neuropsychological and psychosocial assessment data, adapted from the US Health and Retirement Survey to be relevant to the Kenyan context. For four cognitive constructs (orientation, memory, executive functioning, language/fluency) and four psychosocial constructs (depressive symptoms, loneliness, subjective well-being, and life satisfaction), internal consistency reliability was evaluated using McDonald’s omega (ω) and factor structures were evaluated using confirmatory factor analysis. McDonald’s omegas ranged from ω=0.78 to 0.95 for cognitive domains and were above 0.83 for each psychosocial domain, suggesting high reliability. Factor analyses revealed good to excellent fit for most domains (RMSEA<0.095; CFI>0.93; SRMR<0.11); patterns of factor loadings were mostly acceptable (ranging from 0.5 to 0.9), and will be used to refine instruments further. This examination reveals a successful adaptation of neuropsychological and psychosocial assessments in Kenya and provides a reference for other studies seeking to assess the efficacy of adapted instruments.

